# Emergence of *Raoultella ornithinolytica* in human infections from different hospitals in Ecuador with *OXA-*48-producing resistance

**DOI:** 10.3389/fmicb.2023.1216008

**Published:** 2023-08-24

**Authors:** José E. Villacís, Hugo G. Castelán-Sánchez, Jorge Rojas-Vargas, Ulises E. Rodríguez-Cruz, Viviana Albán, Jorge A. Reyes, Pablo M. Meza-Rodríguez, Sonia Dávila-Ramos, Fernando Villavicencio, Margarita Galarza, Monica C. Gestal

**Affiliations:** ^1^Centro de Investigación para la Salud en América Latina (CISeAL), Pontificia Universidad Católica del Ecuador, Quito, Ecuador; ^2^Centro de Referencia Nacional de Resistencia a los Antimicrobianos, Instituto Nacional de Investigación en Salud Pública, “Leopoldo Izquieta Pérez,” Quito, Ecuador; ^3^Programa Investigadoras e Investigadores por México, Grupo de Genómica y Dinámica Evolutiva de Microorganismos Emergentes, Consejo Nacional de Ciencia y Tecnología, México City, Mexico; ^4^Centro de Investigación en Dinámica Celular, Instituto de Investigación en Ciencias Básicas y Aplicadas, Universidad Autónoma del Estado de Morelos, Cuernavaca, Mexico; ^5^Departamento de Microbiología Molecular, Instituto de Biotecnología, Universidad Nacional Autónoma de México, Cuernavaca, Mexico; ^6^Departamento de Ecología Evolutiva, Instituto de Ecología, Universidad Nacional Autónoma de México, México City, Mexico; ^7^Department of Environmental and Occupational Health Sciences, University of Washington, Seattle, WA, United States; ^8^Facultad de Ciencias Químicas, Universidad Central del Ecuador, Quito, Ecuador; ^9^Hospital de Especialidades Eugenio Espejo, Quito, Ecuador; ^10^Department of Microbiology and Immunology, Louisiana State University (LSU), Health Science Center at Shreveport, Shreveport, LA, United States

**Keywords:** *Raoultella ornithinolytica*, antimicrobial resistance (AMR), pangenome analyses, Ecuador (country), whole genome sequencing (WGS)

## Abstract

**Purpose:**

The purpose of this study was to highlight the clinical and molecular features of 13 *Raoultella ornithinolytica* strains isolated from clinical environments in Ecuador, and to perform comparative genomics with previously published genomes of *Raoultella* spp. As *Raoultella* is primarily found in environmental, clinical settings, we focused our work on identifying mechanisms of resistance that can provide this bacterium an advantage to establish and persist in hospital environments.

**Methods:**

We analyzed 13 strains of *Raoultella ornithinolytica* isolated from patients with healthcare associated infections (HAI) in three hospitals in Quito and one in Santo Domingo de Los Tsáchilas, Ecuador, between November 2017 and April 2018. These isolates were subjected to phenotypic antimicrobial susceptibility testing, end-point polymerase chain reaction (PCR) to detect the presence of carbapenemases and whole-genome sequencing.

**Results:**

Polymerase chain reaction revealed that seven isolates were positive isolates for *bla*_OXA–48_ and one for *bla*_KPC–2_ gene. Of the seven strains that presented the *bla*_OXA–48_ gene, six harbored it on an IncFII plasmid, one was inserted into the bacterial chromosome. The *bla*_KPC_ gene was detected in an IncM2/IncR plasmid. From the bioinformatics analysis, nine genomes had the gene *bla*_OXA–48_, originating from Ecuador. Moreover, all *R. ornithinolytica* strains contained the ORN-1 gene, which confers resistance for β-lactams, such as penicillins and cephalosporins. Comparative genome analysis of the strains showed that the pangenome of *R. ornithinolytica* is considered an open pangenome, with 27.77% of core genes, which could be explained by the fact that the antibiotic resistance genes in the ancestral reconstruction are relatively new, suggesting that this genome is constantly incorporating new genes.

**Conclusion:**

These results reveal the genome plasticity of *R. ornithinolytica*, particularly in acquiring antibiotic-resistance genes. The genomic surveillance and infectious control of these uncommon species are important since they may contribute to the burden of antimicrobial resistance and human health.

## 1. Introduction

*Raoultella* species are gram-negative encapsulated bacilli belonging to Enterobacteriaceae (Drancourt et al., 2001). Until 2001, *Raoultella* species were considered part of the genus *Klebsiella*; however, with the current advances in molecular analysis based on *rpoB* sequencing, *Raoultella* was classified as a distinct and unique genus (Boye and Hansen, 2003; Martínez et al., 2004). Species such as *R. terrigena*, *R. planticola*, *R. electrica*, *R. trevisani*, and *R. ornithinolytica* belong to this genus. *R. ornithinolytica* is the most important because it has been associated with symptomatic cases of bacteremia (Kanki et al., 2002; Mau and Ross, 2010; Haruki et al., 2014; Tafoukt et al., 2017; Avellaneda et al., 2020; Wyres et al., 2020), urinary tract infections (García-Lozano et al., 2013; Haruki et al., 2014), joint infections (Beye et al., 2018), and biliary tract infections (Cleveland et al., 2014).

*Raoultella ornithinolytica*, is considered an unusual microorganism in health settings (Reyes et al., 2020). These bacteria are environmental microorganisms found in water, soil, and plants. For many years, this species have not been considered harmful to humans (Kanki et al., 2002). However, it has been found that some *R. ornithinolytica* strains may harbor antibiotic-resistance genes, such as *bla*_NDM–1_, *bla*_OXA–48_, and *bla*_OXA–232_ in the environment (Iovleva et al., 2019), constituting a possible route of transmission and spread of antimicrobial resistance genes (ARGs) through mobile elements (horizontal gene transfer) (Tafoukt et al., 2017; Weng et al., 2020; Zou et al., 2020). A recent report from Croatia, described a case of septicemia in a 64-year-old male patient, caused by *R. ornithinolytica* and *Klebsiella pneumoniae*, both associated with antibiotic resistance and presence of *bla*_OXA–48_ gene, which contributed to severity of infection and course of antibiotic treatment.

The correct identification of *R. ornithinolytica* is one of the main challenges in clinical settings. *R. planticola* and *R. ornithinolytica* share from of 98.3 to 99.5% of their genome content, which leads to form a tight phylogenetic cluster (Bravo, 2006; Walckenaer et al., 2008). Since most clinical laboratories relie on routine automated systems, high rates of misidentification have been reported, (Park et al., 2011; Sȩkowska et al., 2018). These systems are not sensitive enough to discriminate one species from the other through the ornithine decarboxylase (ODC) assay (Alves et al., 2006). In the absence of a robust biochemical assay and specific genetic markers allowing to detect of differences between these two species, the application of proteomic and genomic tools with whole-genome sequencing (WGS) have aided to accurately and rapidly discriminate at the species level. WGS allows us to have a greater number of genetic elements to differentiate them, but also to assess or infer functionality in terms of antimicrobial resistance (Galata et al., 2018).

In this study, we performed a comparative genomic analysis of 13 *R. ornithinolytica* strains isolated from different Ecuadorian hospitals identified by Whole Genome Sequencing (WGS). This comparative analysis revealed that the 13 bacterial strains correspond to different upsurge.

## 2. Materials and methods

### 2.1. Bacterial strains

We received 13 *Raoultella* spp. strains from Hospital Eugenio Espejo–HEE, Hospital Militar–HMI, Hospital Carlos Andrade Marín–HCA, and Hospital Gustavo Dominguez—HGD. These samples were received at the National Reference Center for Antimicrobial Resistance (CRN-RAM) Dr. Leopoldo Izquieta Pérez in Quito. The origin of the samples is shown in Supplementary Table 1. These samples have a similar phenotype to those at the Antimicrobial Resistance Reference Center as part of the national surveillance program which were isolated in Ecuador between November 2017 and April 2018.

The Antimicrobial Resistance (AMR) surveillance, the National Antimicrobial Resistance Reference Center (CRN-RAM) has defined a list of microorganisms with antimicrobial susceptibility patterns that are included in epidemiological surveillance actions in different hospitals in Ecuador. In this case, the hospitals reported the presence of resistance to carbapenems and susceptibility to third-generation cephalosporins, suggesting that this observation could be attributed to resistance mediated by *bla*_OXA–48_ carbapenemase. Consequently, epidemiological alert control measures were implemented, indicating the possible presence of an outbreak with a common source not identified, which was subsequently investigated through phenotypic observation in the laboratory followed by PCR and genomic sequencing. Following the last reported case of *Raoultella ornithinolytica*, hospitals conducted active surveillance for 6 months, during and no further cases were detected.

Isolates were recovered from BD CultureSwab MAXV smears with Stuart liquid medium by plating them on BD Difco nutrient agar and MacConkey agar. Plates were incubated at 37°C for 24 h. Pure colonies were selected for bacterial identification and antimicrobial susceptibility using VITEK^®^2 GN cards and the VITEK^®^2 Compact Microbial Detection System (BioMerieux Inc., France) according to the manufacturer’s recommendations.

### 2.2. Genome sequencing and assembly

Genomic DNA was extracted and purified using the Wizard Genomic Purification Kit (Promega). DNA extracts were used to perform WGS at the Malbran Institute in Argentina by the Illumina Miseq platform using the Nextera XT DNA library preparation kit (Illumina^®^, San Diego, CA, USA). Libraries were generated with a length of 300 bp paired-end reads. The quality control analysis of reads was performed using FastQC software v.011.9 (“Babraham Bioinformatics, 2022–FastQC A Quality Control Tool for High Throughput Sequence Data” n.d.). Subsequently, reads were trimmed using TrimGalore v.0.6.7 (Krueger et al., 2021) with Q>20. The reads were assembled into contigs using SPAdes v.3.13.1 (Bankevich et al., 2012) with the default settings, and the assembly statistics were performed with QUAST v5.0.2 (Gurevich et al., 2013).

### 2.3. Genome identification, annotation, and phylogenomic tree construction

Bacterial identification was performed using the criterion Average Nucleotide Identity (ANI), using PyANI v.0.2.12 with default parameters (Pritchard et al., 2016). Briefly, ANI is defined as the mean nucleotide identity of orthologous gene pairs shared by two microbial genomes considered one genome >95% ANI as the same species (Barco et al., 2020). We retrieved 41 external complete genomes of the genus *Raoultella* from the NCBI portal (retrieved October 9, 2022), as well as 5 genomes of *Klebsiella* as controls for this analysis. A total of 59 genomes were included in the ANI analysis. The assembled genomes were annotated using Prokka v.1.14.6 (Seemann, 2014) and then compared for size and similarity to the reference sequence using BLAST BRIG ring imager v.0.95 (Alikhan et al., 2011).

For phylogenomic reconstruction, a set of 92 bacterial core genes was retrieved from the 59 genomes and aligned using Up-to-date Bacterial Core Gene Software (UBCG) v.3.0 (Na et al., 2018). A maximum-likelihood (ML) tree was estimated with IQ-TREE v2.0.3 (Nguyen et al., 2015) based on 1,000 bootstrap replicates. The five genomes of *Klebsiella* were used as the phylogenetic tree root.

### 2.4. Core and pan-genome analysis

Core and pan-genome analysis were performed using only *R. ornithinolytica* genomes, including our 13 Ecuadorian genomes and 22 complete genomes from NCBI (Supplementary Table 1). The 35 genomes were analyzed using Roary (Page et al., 2015), with 95% identity for blastp and a strict definition of nuclear genome (i.e., 100% of isolates with core genes) (Page et al., 2015).

### 2.5. Antimicrobial susceptibility test, identification, and search for antimicrobial resistance genes and plasmids using bioinformatics

Susceptibility testing was performed using a VITEK-2^®^ AST N272 card (BioMerieux Inc., France). The modified carbapenem inactivation method (mCIM) was performed according to the Clinical and Laboratory Standards Institute (CLSI) to evaluate the presence and expression of enzymatic antimicrobial resistance mechanisms. Molecular analysis by PCR was performed to identify the following ARGs: *bla*_KPC_, *bla*_NDM_, *bla*_VIM_, *bla*_IMP_, and *bla*_OXA–48_, according to previously published protocols described by Poirel et al. (2011) (Table 1). We used the Comprehensive Antibiotic Resistance Database (CARD) in Resistance Gene Identifier (RGI) v.5.1.0 to search ARGs (Alcock et al., 2020), and PLACNETw (Vielva et al., 2017) to search for plasmids, and circular plasmids were visualized through BRING v.0.95, in previously assembled genomes.

**TABLE 1:** Primers for identification of carbapenems.

Gene	Primer	Sequence (5′-3′)	Product size (bp)
*bla*_KPC_	KPC-Fm	CGTCTAGTTCTGCTGTCTTG	798
	KPC-Rm	CTTGTCATCCTTGTTAGGCG	232
*bla*_NDM_	NDM-F	GGTTTGGCGATCTGGTTTTC	621
	NDM-R	CGGAATGGCTCATCACGATC	
*bla*_VIM_	VIM-F	GATGGTGTTTGGTCGCATA	390
	VIM-R	CGAATGCGCAGCACCAG	
*bla*_IMP_	IMP-F	GGAATAGAGTGGCTTAAYTCTC	232
	IMP-R	GGTTTAAYAAAACAACCACC	
*bla*_OXA–48_	OXA48-F	GCGTGGTTAAGGATGAACAC	438
	OXA48-R	CATCAAGTTCAACCCAACCG	

Incompatibility groups (Inc.) and pMLST subtypes were determined *in silico* using PlasmidFinder and pMLST (Carattoli and Hasman, 2020), and ccfind (Nishimura et al., 2017) was used to determine the circulated genome.

### 2.6. Gain and loss genes from ancestral reconstruction in *R. ornithinolytica*

We performed an ancestral reconstruction analysis to estimate genetic gain and loss events. We used the Wagner parsimony model with flexible gain-loss ratios for all lineages and a Poisson distribution at the root of the tree in the program COUNT (Csurös, 2010). The number of orthologs in each genome was determined using Proteinortho, with a threshold of 50% for identity and coverage (Lechner et al., 2011).

## 3. Results

### 3.1. Bacterial strains

From November 2017 to April 2018, a proportional increase in *Raoultella* spp. infections were reported in tertiary hospitals in Quito and Santo Domingo de los Tsachilas, Ecuador; The strains were identified in NRLAR using the VITEK^®^2 GN card compact system (BioMerieux Inc., France) and confirmed as *R. ornithinolytica* by sequencing of the *rpoB* gene.

### 3.2. Genomic features of the *Raoultella ornithinolytica* isolated

**FIGURE 1:**
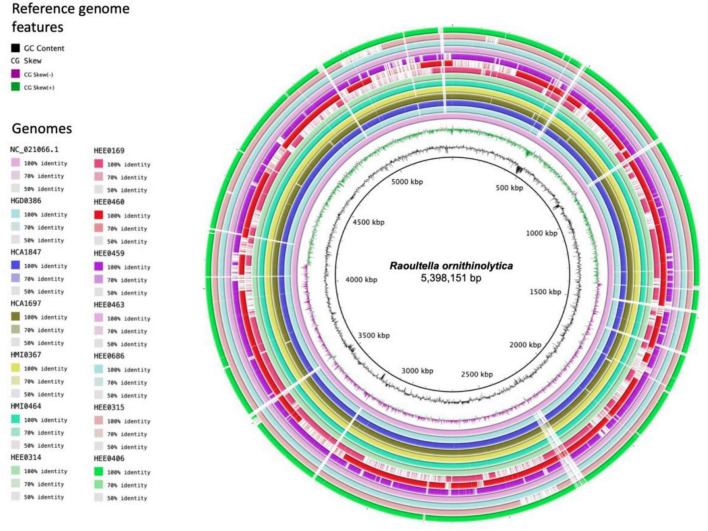
Circular genome representation of *Raoultella ornithinolytica* compared to reference genomes NC_021066.1. The genomes of the 13 strains isolated from hospitals in Ecuador are shown and the predicted coding sequences (CDS) are shown in different colors and the percentage of identity is indicated in the legend.

**FIGURE 2:**
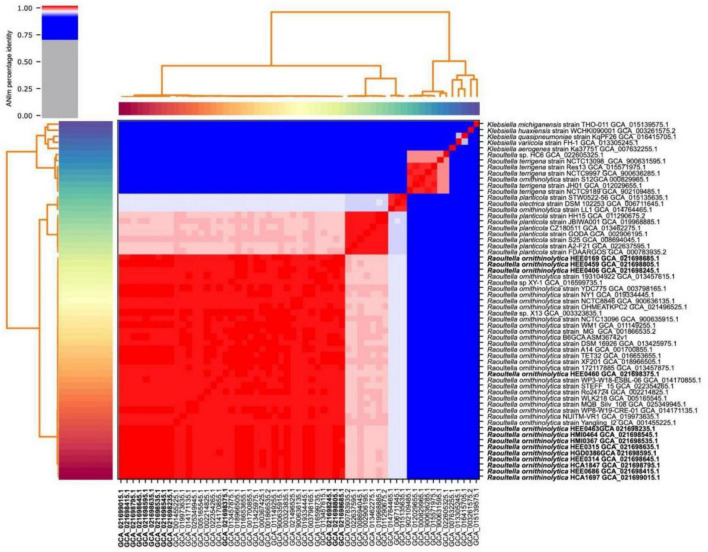
Heatmap of average nucleotide identity (ANI) comparing the 54 genomes of the genus *Raoultella* and 5 of the genus *Klebsiella* used as controls. The heatmap is the product of the matrix generated by pyANI. The red colored cells of the heatmap have >95% sequence similarity, while the blue cells have <95% similarity; once the nucleotide identity reaches 95%, the cells are colored white.

Whole-genome sequencing sequences of the isolated *Raoultella* strains were sequenced individually and then merged. The general characteristics of the 13 assembled genomes are summarized in Table 2. The size of the genomes ranged from 5.5 Mb to 5.9 Mb. The average GC content of each genome was approximately 55.6% (Figure 1). The average number of coding sequences (CDS) in each genome was 5,485 pb, as shown in Table 2. Taxonomic identification was based on average nucleotide identity (ANI), and whole genome comparison results showed that all 13 genomes were nearly 99% concordant with the 22 *R. ornithinolytica* genome references (Figure 2). These results indicate that the isolates from different hospitals in Ecuador corresponded to *R. ornithinolytica*.

**TABLE 2:** Assembly statistics of *Raoultella ornithinolytica* the isolated from hospitals in Ecuador.

Code	Genebank accesión number	Sequence size	Number of contigs	GC content (%)	Median sequence size	Mean sequence size	N50 value	Number of coding sequences
HEE0686	GCF_021698415.1	5,593,786	48	55.6	6024	13822.2	281,078	5,161
HCA1847	GCF_021698795.1	5,593,768	39	55.6	3033	22461.1	400,770	5,153
HMI0464	GCF_021698545.1	5,729,712	52	55.6	17163	36475.0	349,492	5,353
HEE0460	GCF_021698375.1	5,671,979	72	55.7	28061.5	217396	290,183	5,305
HEE0459	GCF_021698805.1	5,715,189	82	55.6	5486	9560.5	195,910	5,368
HEE0406	GCF_021698245.1	5,684,786	67	55.7	10683	32022.8	220,485	5,374
HEE0315	GCF_021698635.1	5,598,932	42	55.6	4345	36781.8	738,672	5,160
HGD0386	GCF_021698595.1	5,582,782	45	55.7	398	21074.5	367,263	5,152
HMI0367	GCF_021698535.1	5,597,243	45	55.6	768	24210.1	401,817	5,105
HEE0314	GCF_021698645.1	5,597,398	43	55.6	16476.0	197527	367,263	5,160
HEE0463	GCF_021698235.1	5,596,758	97	55.6	473	57765.1	393,497	5,153
HEE0169	GCF_021698685.1	5,666,149	75	55.6	7307	22939.2	215,878	5,354
HCA1697	GCF_021699015.1	5,927,737	70	55.3	570	37620.9	363,932	5,590

**FIGURE 3:**
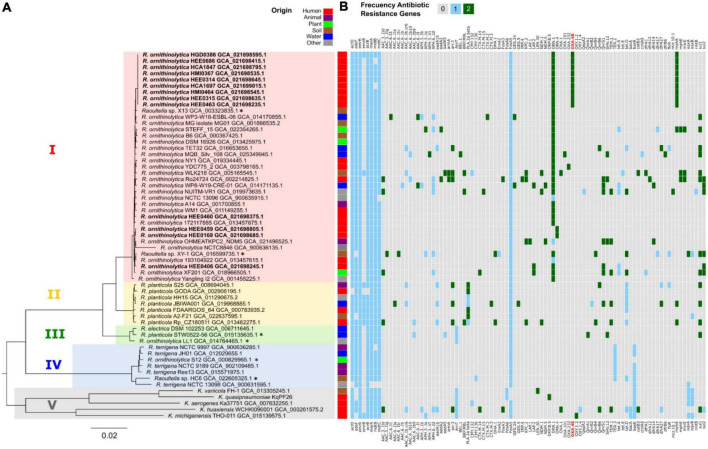
Phylogeny and resistance genes. **(A)** Phylogenetic tree of the *Raoultella* genus based on the 92 individual genes analyzed with 92 genes from UBCG and maximum likelihood with IQtree. Clades are shown, clade I in red corresponds to *R. ornithinolytica*, clade II in yellow corresponds to *R. planticola*, clade III in green corresponds to *Raoultella* spp., clade IV in blue corresponds to *R. terrigena*, and clade V in brown corresponds to *Klebsiella* spp. Asterisks indicate genomes with failed taxonomy checks in the NCBI portal. The *R. ornithinolytica* genomes in this study are shown in bold, with a clade in the upper part of the tree that groups nine genomes from Hospital Carlos Andrade Marín, Hospital Militar, Hospital Eugenio Espejo, and Hospital Gustavo Dominguez. The origin of each strain is shown in different colors. **(B)** Heatmap of resistance genes in *Raoultella* genus using Resistance Gene Identifier (RGI). The *R. ornithinolytica* genomes in this study show a pattern of resistance to *bla*_OXA–48_ (in red), *blaOKP-5*, and *mphA* gene, whereas the other genomes within the genus show no resistance to *bla*_OXA–48_.

A phylogenetic tree was constructed using a set of 92 bacterial conserved genes to infer the phylogenetic relationship between our 13 genomes of *R. ornithinolytica*, and other 46 complete genomes of the genera *Raoultella* and *Klebsiella* retrieved from the NCBI portal. There are five main clades, as shown by the different colors in Figure 3. Clade I for *R. ornithinolytica*, Clade II for *R. planticola*, Clade III for *Raoultella* spp., Clade IV for *R. terrigena*, and Clade V for *Klebsiella* spp. In Clade I, nine out of thirteen Ecuadorian genomes of *R. ornithinolytica* were grouped together in a well-supported subclade, suggesting the idea of a common origin. The remaining four genomes, came from a single healthcare setting and seem to be dispersed throughout different subclades within Clade I, which suggests independent introduction events. In contrast, strains scattered in the tree may not belong to the same outbreak despite the geographical distance between the hospitals.

### 3.3. Pan-genome comparative analyses of *R. ornithinolytica* genomes

**FIGURE 4:**
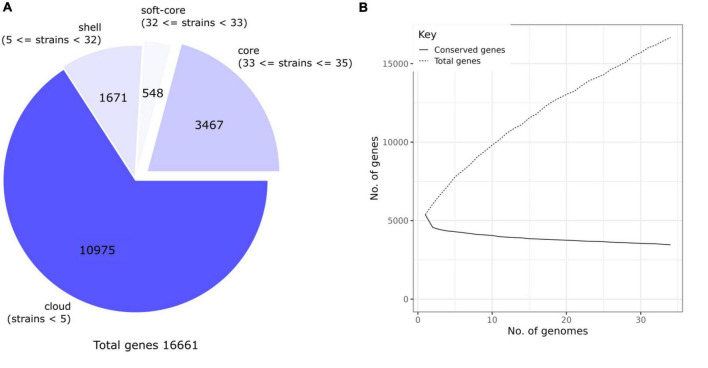
Core and pan-genome analyses of *Raoultella ornithinolytica*. **(A)** Pan-genome pie chart created with the Roary software. The gene content in the nucleus, soft nucleus, shell, and cloud is represented by the pie. The cloud contains the most genes, while the core genome contains the fewest genes. **(B)** The Heap’s Law diagram is plotted. The diagram shows the conserved genes relative to the total number of genes in 35 *R. ornithinolytica* genomes. The pan-openness of the genomes reflects the diversity of the gene pool within the *R. ornithinolytica* genomes.

The pan-genome of the 35 genomes analyzed is shown in Figure 4. A total of 16,661 gene clusters (orthologs) were found, of which 3,467 genes (27.77%) were assigned to the core genome, 548 to the soft-core genome (3.29%), and 10,975 to the unique genome (65.87%) (Figure 4A). To investigate if the *R. ornithinolytica* pan-genomic is open, which it will suggest and increase in genome size due to the addition of new genomes, Heap’s law was applied (Figure 4B). The trend of Heap’s law diagram for the pangenome shows gradual expansion due to the addition of new genomes, with the slope continuing to increase. The curve shows the relationship between the core genome and the number of genomes, which decreased and did not exceed 5,000 genes. Thus, the pangenome of *R. ornithinolytica* is reflected in the openness of the pan-genome, which could indicate high genomic plasticity of this species and, thus, the greater potential of adaptability.

### 3.4. Antimicrobial resistance genes and plasmids

**FIGURE 5:**
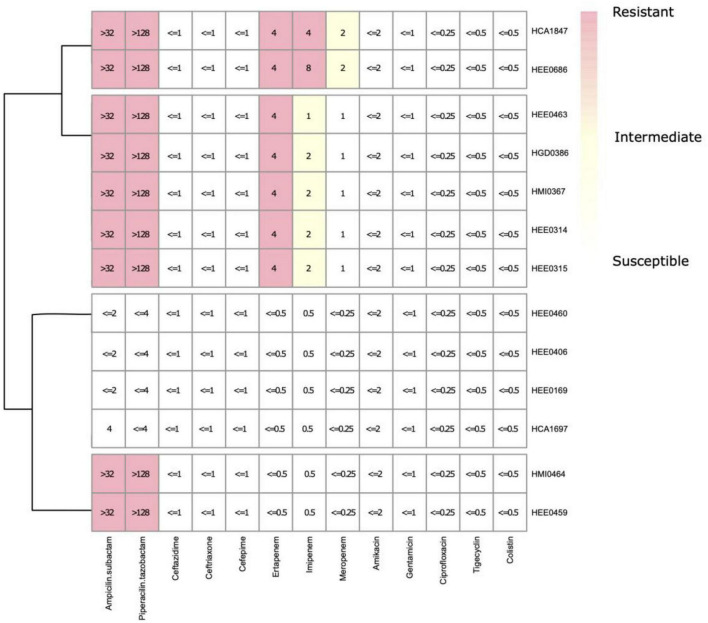
Experimental minimum inhibitory concentrations (MICs) distribution of the isolates of *Raoultella ornithinolytica* from Ecuador.

The results of antimicrobial susceptibility testing showed three different resistance phenotypic patterns (Figure 5 and Supplementary Table 2). The first includes resistance to ampicillin/sulbactam (SAM), piperacillin/tazobactam (TPZ), ertapenem (ETP), imipenem (IMP), and intermediate resistance to meropenem (MEM). The second pattern shows, resistance to SAM, TPZ, and ETP, and intermediate resistance to IMP. And the third one shows the resistance to SAM and TPZ exclusively. No specific antimicrobial resistance patterns were observed within hospitals. Therefore, the phenotypic antimicrobial resistance patterns observed in the first, second, and third groups have no consistent association with any particular hospital, as described above.

Seven *R. ornithinolytica* strains showed interesting patterns of at least two out of three carbapenems testing resistance, but appearing susceptible to third and fourth-generation cephalosporins (ceftazidime, ceftriaxone, and cefepime), which is a consistent phenotypic profile, indicating the possible presence of a *bla*_OXA–48_ carbapenemase, which was confirmed by them CIM assay and PCR (Figure 5).

In addition, bioinformatic analysis of antimicrobial resistance was performed using all the genomes collected using RGI software (Alcock et al., 2020; Figure 3). The heatmap results showed bit scores using BLAST with RGI values Perfect (green), Strict (blue), and Losse (gray) (Figure 3B). Among the genes predicted with a perfect bitscore with RGI analysis was the ORN-1 gene, which encoded for an Ambler class A beta-lactamase conferring resistance to penicillins and cephalosporins (Walckenaer et al., 2008). ORN-1 gene was found in all of our isolates and in 14 of the *R. ornithinolytica* genomes retrieved from NCBI for comparison purposes. The Ecuadorian genomes grouped in Clade I (Figure 3A) positive for ORN-1, also carried the *mphA* gene, which confers resistance to macrolides (Li et al., 2022) and the *bla*_OXA–48_ gene, which confers resistance to carbapenems; however, only seven of these isolates expressed a phenotypic resistance to carbapenems.

In addition, genes conferring resistance to aminoglycosides, beta-lactams, sulfates, tetracyclines, and dihydrofolate, such as *acrB*, *acrD*, *mdtC*, *marA*, *mdtB*, and *adeF* were present in all the Ecuadorian isolates. Nevertheless, the diversity of ARGs showed among the Ecuadorian isolates was lower when compared with the ARGs diversity from other *R. ornithinolytica* strains (Figure 3). Only one Ecuadorian strain (HEE0406) harbored additional antimicrobial resistance genes (*SHV1.2*, *tetA*, and *fosA*).

We have identified genes associated with antibiotic resistance in 58 isolates, and the gene *fosA* was also found in 56 isolates.

**FIGURE 6:**
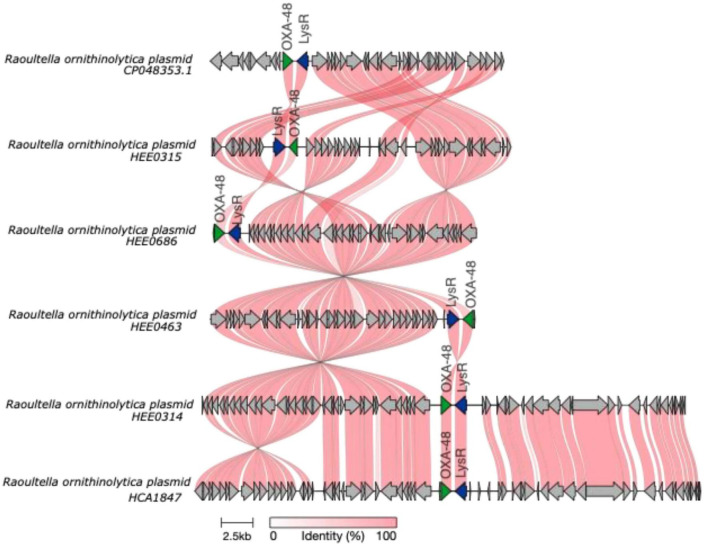
Schematic representation was made using clinker (Gilchrist and Chooi, 2021) of *bla*^*OXA*−48^ plasmids in *Raoultella ornithinolytica* genomes obtained from the clinical strains isolated in Ecuador. The frames for the genes are shown as arrows; in green is the *bla*^*OXA*−48^ gene, flagged by the *lysR* gene.

Finally, plasmid analyses revealed that the Ecuadorian *R. ornithinolytica* strains contained segments of plasmids. The partial plasmid containing the *bla*_OXA–48_ gene (22 Kb), is found in HEE0315, HEE0686, HEE0463, HEE0314, and HCA1847 strains (Figure 6). Unfortunately, the complete plasmid could not be obtained, but based on previous literature and based on our knowledge, we believe that this gene could be responsible for the resistance in *R. ornithinolytica*.

### 3.5. Gain and loss of genes from ancestral reconstruction in *R. ornithinolytica*

**FIGURE 7:**
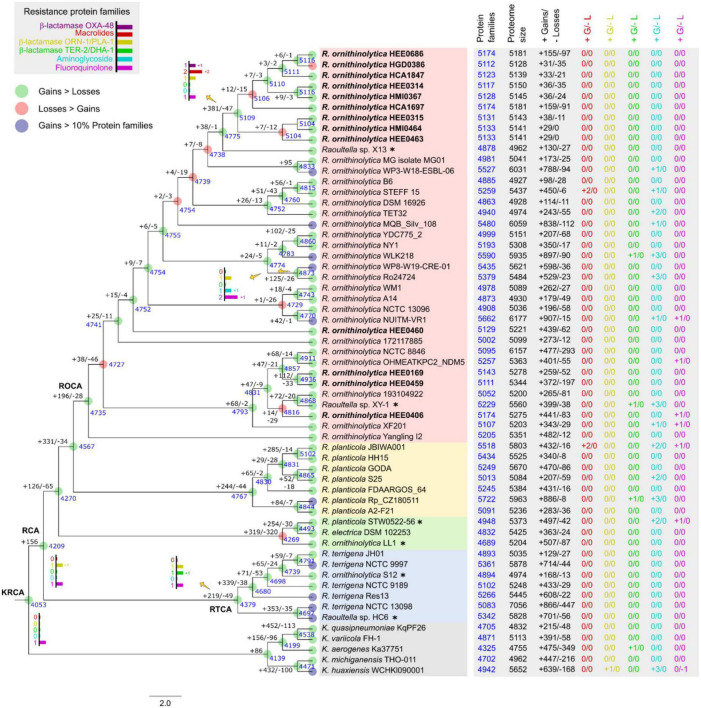
Ancestral reconstruction of genomes of *Raoultella ornithinolytica* using COUNT software. The number of gene family gains (“ + ”) and losses (“–”) were calculated using Wagner’s parsimony are presented in each branch, and the number of gene families are shown in blue in the corresponding branch. The tree topology is based on the maximum likelihood in Figure 3. The bar charts show the numbers of gained/losses of the protein families related to resistance observed in common ancestors. Nodes with more gain events are indicated in green circles; nodes with more loss events are in red circles; nodes with significant genome expansion (>10% gains) are indicated in blue circles. The table shows number features of the selected genomes: protein families, proteome size, total gains/losses events, and gains/losses events of the protein families related to resistance observed in each terminal node. KRCA, *Klebsiella/Raoultella* common ancestor; RCA, *Raoultella* common ancestor; RTCA, *R. terrigena* common ancestor; ROCA, *R. ornithinolytica* common ancestor.

Since our results revealed that the pangenome of *R. ornithinolytica* is open, the gain or loss of genes during their evolutionary history is expected. To this end, we performed an ancestral reconstruction analysis using WGS (Figure 7). The genomes examined shared over 4,000 protein families with their common ancestor *Klebsiella/Raoultella* (KRCA), and the reconstruction analysis predicted more gain than loss events.

Our analysis revealed that six protein families associated with antibiotic resistance were acquired from five predicted common ancestors (Figure 7). We hypothesize that the most remote ancestor, KRCA, harbor genes that encode an efflux pump providing resistance to fluoroquinolones (Poole, 2000), and that these genes could have been transmitted as orthologs to the analyzed genomes, except for the *K. huaxiensis* WCHKl090001 which lost that protein family. Our second hypothesis is that the common ancestor of *Raoultella* (RCA) acquired genes associated with β-lactamase resistance ORN-1/PLA-1, which were not subsequently lost. The common ancestor of *R. terrigena* (RTCA) acquired genes conferring β-lactamase resistance TER −2/DHA −1 and passed vertically to strains in this group.

In addition, the common ancestor of *R. ornithinolytica* WP8-W19-CRE-01 and *R. ornithinolytica* Ro24724 acquired genes for a pentapeptide repeat protein and aminoglycoside acetyltransferases related to fluoroquinolone and aminoglycoside resistance, respectively (Vetting et al., 2006; Reeves et al., 2020). Even though they have been isolated from different sources in China. Strain WP8-W19-CRE-01 was obtained from wastewater, whereas strain Ro24724 was obtained from necrotic tissue.

The last common ancestor with resistance protein families was the ancestor of clinical strains HEE0686, HGD0386, HCA1847, HEE0314, HMI0367, HCA1697, HEE0315, HMI0464, and HEE0463. This ancestor acquired two protein families related to the multidrug efflux pump *Tap*, and a hypothetical protein related to macrolide resistance. This ancestor may also have gained OXA-48, which was only present in this clade (Figure 3). In general, there are other gains/losses occurring in the genomes, but these do not present traceable ancestors in the tree because changes may have occurred recently.

## 4. Discussion

*Raoultella ornithinolytica* is a gram-negative bacterium isolated from various environments, including water and soil, as well as from various animals, including birds, humans, and other mammals (Seng et al., 2016). *R. ornithinolytica* has been shown to cause human diseases; however, its incidence may be underestimated because of the high misidentification rate of automated systems based on phenotypic characteristics (Sȩkowska et al., 2018). The *Raoultella* genus has been identified as *Klebsiella oxytoca* (Walckenaer et al., 2008; Park et al., 2011; Sȩkowska et al., 2018).

To overcome these limitations, Whole Genome Sequencing (WGS) has been used to identify *R. ornithinolytica* with greater accuracy, allowing the identification of genes that may be important for bacterial metabolism and pathogenicity (Matar et al., 2020). In Ecuador, WGS was used to identify *Raoultella* isolates, and the results showed that 13 bacteria isolated were *R. ornithinolytica.* The ANI, and taxonomic position resulted in a phylogenetic tree analysis with 92 marker genes, which showed that the strains belonged to *R. ornithinolytica* (Matar et al., 2020).

In the phylogenetic tree, nine strains from Ecuador formed a cluster, suggesting that these strains had greater genomic and phenotypic similarities than the other four strains scattered independently in the tree. This suggests that the first nine strains belonged to a single outbreak. The genomic characteristics of the 13 isolated genomes found in Ecuadorian hospitals are summarized in Table 2. The genomes reported in this study had a 1% size difference average variation among themselves, suggesting lost and gain events.

The phylogenetic clustering of strains within the observed phylogenetic tree indicates a potential localized transmission event or a shared exposure to a common source of infection, despite being identified in different hospitals. The clustering pattern strongly suggests the likelihood of an outbreak, which appears to have occurred within a specific time frame spanning from November 2017 to April 2018. Following this period, an active surveillance program was implemented for an additional 6 months, during which screening efforts were undertaken to identify strains exhibiting the same phenotypic resistance pattern for subsequent genomic sequencing. However, no strains with the identified resistance pattern were detected beyond this surveillance period.

On the other hand, the core genome was found to include 33% of the pangenome of all the *R. ornithinolytica* assemblies, using a high identity value (≥95% identity). In other species, small pan-genome size indicates a process of speciation/adaptation (Zhang et al., 2018). As more genomes were examined, the total number of genes observed in the analysis also increased, as shown in Figure 4B. However, this pan-genomic feature allows different strains to survive in various environmental niches (Medini et al., 2005; McInerney et al., 2017). The core genome of *R. ornithinolytica* is small compared to close bacteria, such as *Streptococcus. pneumoniae* (55.06%), *Staphylococcus aureus* subsp. *aureus* (75.23%), *Escherichia coli* (53.34%), *Salmonella enterica* subsp. *enterica* (71.23%), and *Acinetobacter baumannii* (61.36%) (Park et al., 2019).

Furthermore, results of antibiotic resistance detected by PCR showed seven positive isolates for *bla*_OXA–48_ and one for *bla*_KPC–2_, while bioinformatic analysis revealed nine genomes from Ecuador. Carbapenem resistance in aquatic environments is caused by *bla*_NDM_, *bla*_OXA–48_, and *bla*_KPC–2_ in *R. ornithinolytica* (Hajjar et al., 2020). The emergence of carbapenem-resistant *K. pneumoniae* strains in Ecuador have increased recently. Indeed, OXA-48 carbapenemase has been detected in *K. pneumoniae* isolates from clinical samples (Reyes et al., 2020). In another case, a genome of *R. ornithinolytica* from Ecuador was reported to harbor the *bla*_OXA–48–_*_like_* gene (Reyes et al., 2020). Given the potential for multidrug resistance and the ability of *R. ornithinolytica* to survive in different environmental niches, it is important to continue surveillance to better understand its potential to spread antibiotic resistance.

We conducted ancestral reconstruction on all the genes in the *Raoultella* genome and identified genes that are unique to this species. In particular, we paid close attention to resistance genes to determine if they were present in the ancestral lineage. However, our analysis provides evidence to suggest that some resistance genes were ancestral, but many others are relatively recent in origin. An instance of this is the OXA-48-β-lactamase family, ORN-1/PLA-1, and fluoroquinolone resistance genes found in *Raoultella* genomes, which are likely acquired through horizontal gene transfer and not ancestral. This is consistent with the open pangenome of *R. ornithinolytica*. Ancestral reconstruction analysis reveals that each species in the genus has gained genes recently, potentially via gene transfer, indicating a worrying trend of rapid acquisition and dissemination of antibiotic resistance genes.

A gene harbored by the common ancestor of the *R. ornithinolytica* hospital clade, important for antibiotic resistance, was the IS5 family transposase IS4811, also found in some *Klebsiella* and *R. terrigena* assemblies. This transposase is important for the transfer of genetic material between bacteria, and it can contribute to the spread of antibiotic resistance genes among bacterial populations (Vandecraen et al., 2017).

Also, this ancestor contains the Multidrug efflux pump *Tap*, and a gene encoding for a hypothetical protein related to resistance to macrolides, suggesting that these genes are important for antibiotic resistance in this clade of bacteria. Multidrug efflux pump *Tap* and the hypothetical protein genes were also present in other genomes, such as *R. ornithinolytica* STEFF_15, *R. planticola* JBIWA001, and *R. planticola* Rp_CZ180511, suggesting that they may have been horizontally transferred between different *Raoultella* species.

The presence of the β-lactamase ORN-1/PLA-1 protein family since the *Raoultella* common ancestor, and its acquisition by *K. huaxiensis* WCHKl090001, suggests that this gene family is important for the survival of these bacteria in the presence of β-lactam antibiotics.

The β-lactamase TER-2, a β-lactamase DHA-1, is common to all *R. terrigena*, and according to Figure 7, it has orthologs in *Raoultella* sp. XY-1, *R. planticola* Rp_CZ180511 and *K. aerogenes* Ka37751. These genes likely provide these bacteria with resistance to β-lactam antibiotics, which are commonly used to treat bacterial infections. The acquisition of these resistance genes may have facilitated the spread of antibiotic resistance among different bacterial species, which is a major public health concern (Pérez-Llarena et al., 2014).

The only common ancestor with an aminoglycoside resistance family was found between the *R. ornithinolytica* WP8-W19-CRE-01 and *R. ornithinolytica* Ro24724 genomes, including the gene for Aminoglycoside N(6′)-acetyltransferase type 1. The other aminoglycoside resistance gene families (*aac*, *aad*, *arm*) were gained by the strains.

The common ancestor for *Raoultella* and *Klebsiella* has one fluoroquinolone resistance protein family, encoding for Efflux pump periplasmic linker *BepF*, related to this activity (Li and Nikaido, 2009). All the reported *Raoultella* genomes from Ecuador keep these genes. The *Raoultella sp*. XY-1 gained two copies of this gene and, like some other later characters, acquired the repeat protein Qnr (Figure 3).

In conclusion, continued monitoring and research of *R. ornithinolytica* are necessary to better understand its potential for multidrug resistance. This study highlights the importance of using Whole Genome Sequencing as a valuable tool in identifying *R. ornithinolytica* with greater accuracy and overcoming the limitations of automated systems based on phenotypic characteristics. Finally, the study emphasizes the emergence of multidrug-resistant enterobacteria, including *R. ornithinolytica*, which raises concerns regarding the spread of antibiotic resistance and the potential for horizontal gene transfer.

## Data availability statement

The datasets presented in this study can be found in online repositories. The names of the repository/repositories and accession number(s) can be found in the article/Supplementary material.

## Author contributions

JV, HC-S, JR-V, and MCG: conceptualization. HC-S, JR-V, VA, UR-C, PM-R, FV, and MCG: methodology. JV, HC-S, MG, JR-V, and VA: writing–original draft preparation. JV, HC-S, JR-V, UR-C, VA, MG, SD-R, and MCG: writing–review and editing. HC-S, JR-V, UR-C, and VA: formal analysis. JV, HC-S, JR-V, SD-R, MCG, and MG: supervision and review. JV and MCG: project administration and funding acquisition. All authors have read and agreed to the published version of the manuscript.
